# Total syntheses of highly oxidative *Ryania* diterpenoids facilitated by innovations in synthetic strategies

**DOI:** 10.3762/bjoc.21.198

**Published:** 2025-11-19

**Authors:** Zhi-Qi Cao, Jin-Bao Qiao, Yu-Ming Zhao

**Affiliations:** 1 Key Laboratory of Applied Surface and Colloid Chemistry of MOE & School of Chemistry and Chemical Engineering, Shaanxi Normal University, Xi’an, 710119, China

**Keywords:** natural products, *Ryania* diterpenoids, synthetic strategy, total synthesis

## Abstract

Innovations in synthetic methods and strategic design serve as the primary driving forces behind the advancement of organic synthetic chemistry. With rapidly evolving organic synthesis technologies, a diverse array of novel methods and sophisticated strategies continues to emerge. These approaches not only complement and synergize with one another but also significantly enhance synthetic efficiency, reduce costs, and provide robust solutions to challenges encountered in the synthesis of complex molecular architectures. *Ryania* diterpenes are natural products characterized by intricate structures and high oxidation states. Biological studies have revealed that the family member ryanodine has a specific regulatory effect on myocardial calcium ion channels (PyR). Since only a limited number of compounds have been reported to act by modifying this receptor, ryanodine and its derivatives are potential therapeutic agents for treating cardiovascular diseases. This article focuses on reviewing the efficient application of ring-construction methods and synthetic strategies in the total synthesis of highly oxidized *Ryania* diterpenoid natural products, emphasizing the pivotal role of novel synthetic methods and strategic innovations.

## Introduction

Organic synthesis, as a cornerstone of chemical research, is dedicated to constructing complex natural products or target molecules from simple and readily available starting materials via a series of precise and efficient chemical reactions. This field serves not only as a vital tool for molecular structure validation and the discovery of new reaction mechanisms but also as a fundamental driving force behind advances in related disciplines such as pharmaceutical science. Throughout this endeavor, innovations in methods and strategies function as an engine, consistently pushing the boundaries of the discipline. From the early synthesis of simple molecules to the current precise assembly of complex natural products and functional materials, the iteration of methods and optimization of strategies have always been key to breaking through synthetic bottlenecks.

Since Nobel laureate E. J. Corey proposed the revolutionary concept of “retrosynthetic analysis” [1], the design of synthetic strategies has built upon this core intellectual framework: starting from the target molecule, a stepwise deconstruction guided by reverse logic leads to a series of structurally simple and readily accessible precursor compounds [2]. Based on this philosophy, chemists have developed a variety of classical synthetic strategies to address target molecules with diverse structural features. For instance, “divergent synthesis” employs a universal chiral advanced intermediate, systematically deriving multiple structurally related natural products through functional group transformations and oxidation-state adjustments [3–4]. This approach efficiently constructs compound family libraries, greatly facilitating drug screening and structure–activity relationship (SAR) studies. Conversely, the “biomimetic synthesis” strategy mimics nature’s enzyme-catalyzed pathways to construct target molecules in the laboratory, offering milder reaction conditions and more concise synthetic steps, while demonstrating excellent atom economy and step economy [5–7].

These powerful and diverse methods continuously drive synthetic chemistry forward through deep integration and synergistic application. This article focuses on the total synthesis of highly oxidized *Ryania* diterpenoid natural products, systematically reviewing the synthetic strategies and ring-construction methods employed therein while providing an in-depth analysis of the innovation of classical methods, the application of emerging technologies, and the enhancements in synthetic efficiency achieved through multi-strategy integration. The aim is to offer readers a clear understanding of the developmental trajectory and future trends in the total synthesis of natural products from this family.

**Scheme 1:**
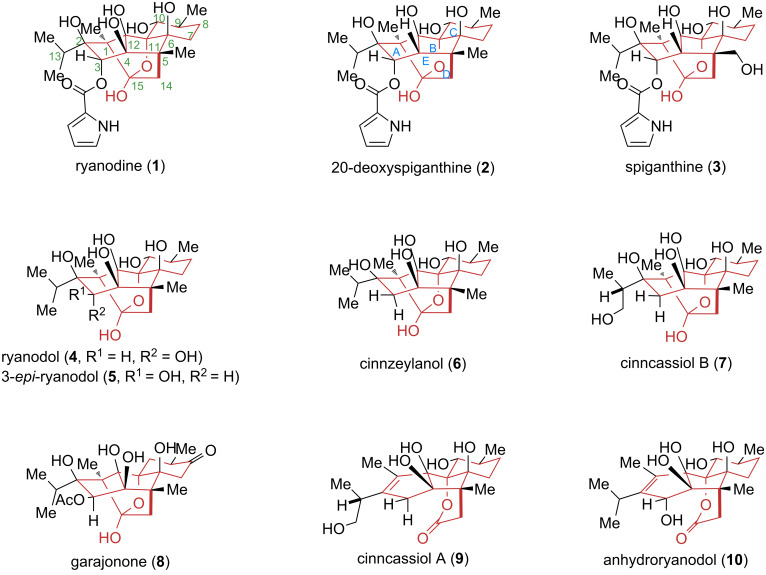
Representative *Ryania* diterpenoids and their derivatives.

Natural products derived from the *Ryania* genus comprise a class of structurally intricate polycyclic diterpenoids isolated from the Central and South American shrub *Ryania speciosa* (Scheme 1) [8–14]. Research on these compounds dates back to 1943 when the American pharmaceutical company Merck developed a novel insecticide, Ryanex, from the stems and leaves of the plant. In 1948, Folkers and colleagues reported the isolation of the first bioactive member of this family – ryanodine (**1**) [8]. Due to limitations in technical capabilities at the time, its absolute configuration remained undetermined. Over the subsequent two decades, its hydrolysis product ryanodol (**4**) and several structurally related analogs were isolated sequentially [9–14]. The absolute configurations of both **1** and **4** were ultimately elucidated in 1968 by Wiesner and co-workers using a combination of chemical degradation and X-ray crystallography [14]. Notably, in 2016, Inoue’s group at the University of Tokyo reconciled discrepancies through comparative analysis of experimental and natural product data, confirming the correct structure of ryanodol to be 3-*epi*-ryanodol (**5**), thereby revising the previously accepted configuration [15]. Since then, numerous analogs based on the ryanodol scaffold have been identified and characterized. As of now, 18 natural products from this family have been successfully isolated and structurally established [16–18].

Structurally, ryanodine (**1**) and related diterpenoid natural products feature a 6-5-5-5-6 pentacyclic core skeleton containing 11 stereocenters, eight of which are quaternary carbons. A key structural feature is the assembly of a polycyclic cage-like framework via multiple C–C and C–O bonds, incorporating a labile hemiketal moiety. Furthermore, the presence of multiple oxygenated quaternary carbons classifies these molecules among the most highly oxidized diterpenoid natural products reported in the literature.

In terms of biological activity, ryanodine (**1**) exhibits high specificity and regulatory effects on ryanodine receptors (RyRs) [19–20]. It is among the few small organic molecules identified to date that can modulate these receptors. Dysfunctions of RyRs are closely associated with various diseases: mutations in RyRs can cause genetic disorders such as malignant hyperthermia and central nervous system disorders; altered expression of RyR2 and RyR3 is linked to the pathogenesis of neurodegenerative diseases like Alzheimer’s disease. Notably, as a critical calcium ion channel in cardiac muscle, RyRs are intimately involved in the development and progression of cardiovascular diseases [21–24]. Additionally, compounds such as cinnzeylanol (**6**), cinncassiol B (**7**), and cinncassiol A (**9**) exhibit various potential biological activities, including insecticidal, ion channel modulatory, and immunosuppressive effects [25–29].

## Review

### Synthetic research on *Ryania* diterpenoid natural products

*Ryania* diterpenoids have garnered sustained interest in the synthetic chemistry community due to their complex, unique molecular structures and potential biological activities. This has motivated extensive research that has led to notable advances. This review summarizes total synthesis efforts of various research groups on *Ryania* diterpenoid natural products, focusing on strategic methods for assembling the 6-5-5-5-6 pentacyclic core skeleton. Furthermore, it examines the integration and synergy of multiple synthetic approaches in constructing this intricate framework, emphasizing their value in addressing highly challenging synthetic endeavors.

#### Deslongchamps’ total synthesis of ryanodol (**4**) and 3-*epi*-ryanodol (**5**)

**Scheme 2:**
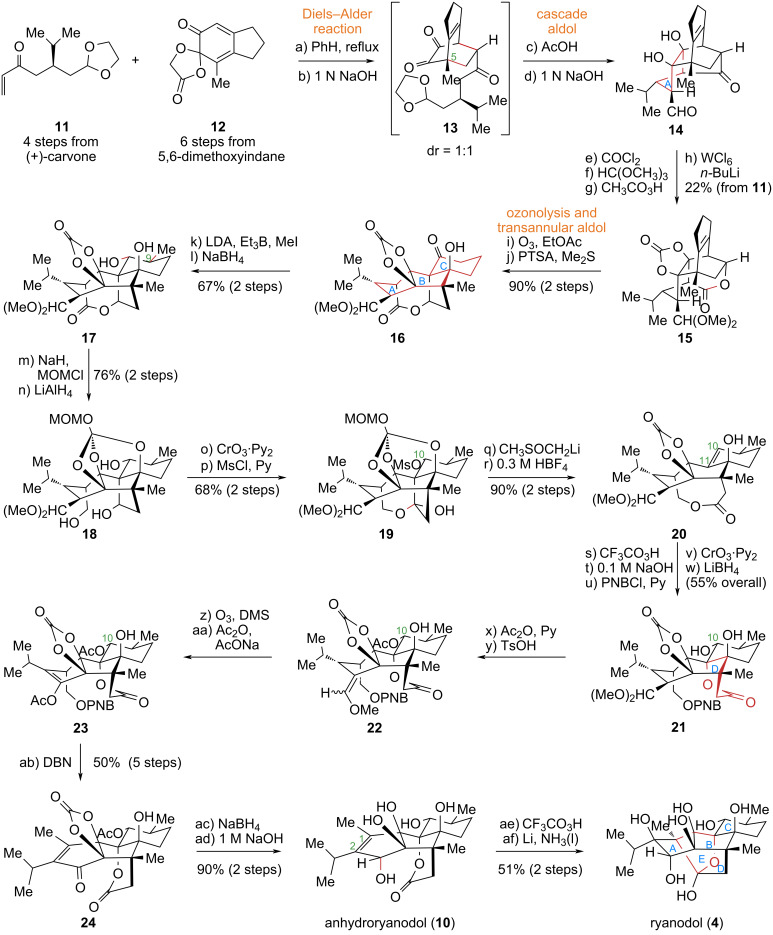
Deslongchamps’s total synthesis of ryanodol (**4**).

In 1979, the Canadian organic chemist Deslongchamps, after a decade of dedicated research, successfully achieved the first total synthesis of the non-naturally produced ryanodol (**4**) and its dehydrated derivative, anhydroryanodol (**10**) [30] (Scheme 2). Given the highly complex fused-ring system and the stereochemical challenges posed by multiple chiral centers, the author utilized the Diels–Alder reaction, a prominent representative of pericyclic reactions [31–44], to control the formation of the crucial C5 chiral center precisely. Subsequent oxidative cleavage of the carbon–carbon double bond introduced in the Diels–Alder reaction, followed by an intramolecular aldol reaction, efficiently constructed the ABC tricyclic core skeleton of the target molecule. This achievement transformed a simple monocyclic precursor into a complex fused-ring skeleton, vividly demonstrating the application value of the multi-reaction synergistic strategy in natural product synthesis.

The specific synthetic route is as follows: Starting from the chiral compound (*S*)-carvone, four simple transformations yield the enone intermediate **11**. This intermediate undergoes an intermolecular [4 + 2] cycloaddition with diene **12**, generating two sets of regioselective products in an approximate ratio of 1:1. The product with the correct relative configuration undergoes hydrolysis of its spirocyclic lactone moiety under basic conditions to yield **13**, establishing the critical C5 chiral center. Under acidic conditions, intermediate **13** undergoes ketal deprotection followed by successive intramolecular aldol reactions, smoothly constructing the A ring to afford compound **14**. Subsequent protection of the vicinal diol and aldehyde functionalities in **14** provides an intermediate that, after Baeyer–Villiger oxidation and subsequent tungsten-promoted reverse epoxidation, forms lactone **15**. Ozonolysis of **15** cleaves the double bond, and a subsequent transannular aldol reaction efficiently assembles the B and C rings, yielding the ABC tricyclic core **16**. Further manipulations included the introduction of a methyl group at C9, adjustments of the oxidation state, and the installation of a mesylate group at C10, leading to compound **19**. This intermediate is converted to lactone **20** via base-promoted Grob fragmentation followed by acid-mediated MOM deprotection. Epoxidation of the C10–C11 double bond in **20**, lactone hydrolysis-promoted epoxide ring opening, and inversion of the C10 hydroxy configuration, yield the key intermediate **21**, thereby completing the construction of the D ring. Adjustments of functional groups and oxidation states at multiple sites then afford anhydroryanodol (**10**). Finally, epoxidation of the C1–C2 double bond followed by Li/NH_3_-promoted reductive cyclization constructs the E ring of the molecular core, successfully completing the first asymmetric total synthesis of ryanodol (**4**) in 41 steps.

To elucidate the role of the C15 hemiacetal hydroxy group in ryanodine (**1**)-type diterpenoid natural products in binding to ryanodine receptors, the authors initially proposed reducing the lactone moiety in anhydroryanodine (not shown) to the corresponding hemiacetal. However, common reducing agents proved ineffective for lactone reduction. Leveraging previous findings, the authors implemented an alternative strategy involving two sequential intramolecular reductive cyclizations to invert the configuration of the C3 secondary hydroxy group, successfully achieving the conversion of ryanodol (**4**) to 3-*epi*-ryanodol (**5**) and 3-*epi*-ryanodine (**30**) [45].

**Scheme 3:**
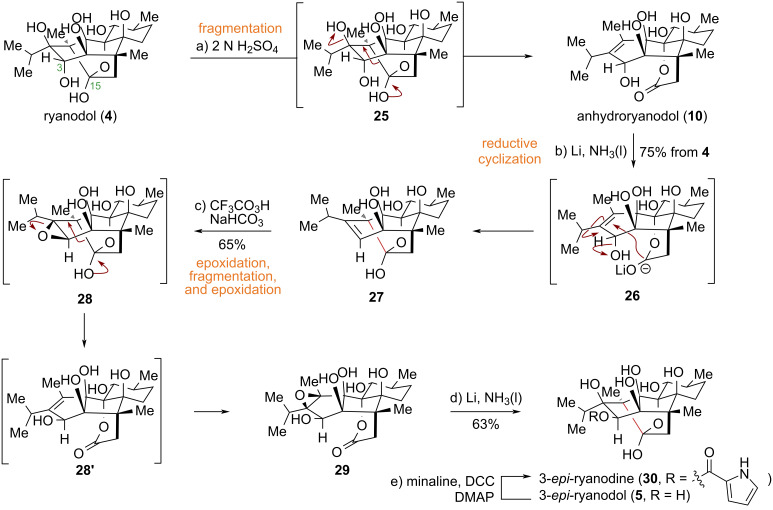
Deslongchamps’s total synthesis of 3-*epi*-ryanodol (**5**).

The specific synthetic route is as follows (Scheme 3): Beginning with ryanodol (**4**), an acid-promoted fragmentation yields anhydroryanodol (**10**). Subjecting compound **10** to Li/NH_3_ conditions induces the first intramolecular reductive cyclization, affording hemiacetal **27**. This intermediate is then transformed via a one-pot sequence involving epoxidation, fragmentation, and re-epoxidation to give epoxide **29**. A second intramolecular reductive cyclization of **29** under Li/NH_3_ forms the intramolecular oxa-bridged ring, culminating in the first total synthesis of 3-*epi*-ryanodol (**5**) and 3-*epi*-ryanodine (**30**). The subsequent biological evaluation revealed that **30** possesses only 1% of the affinity of ryanodine (**1**) for ryanodine receptors (RyRs).

#### Inoue’s total synthesis of ryanodine, ryanodol, 3-*epi*-ryanodol, cinnzeylanol, and cinncassiols A,B

**Scheme 4:**
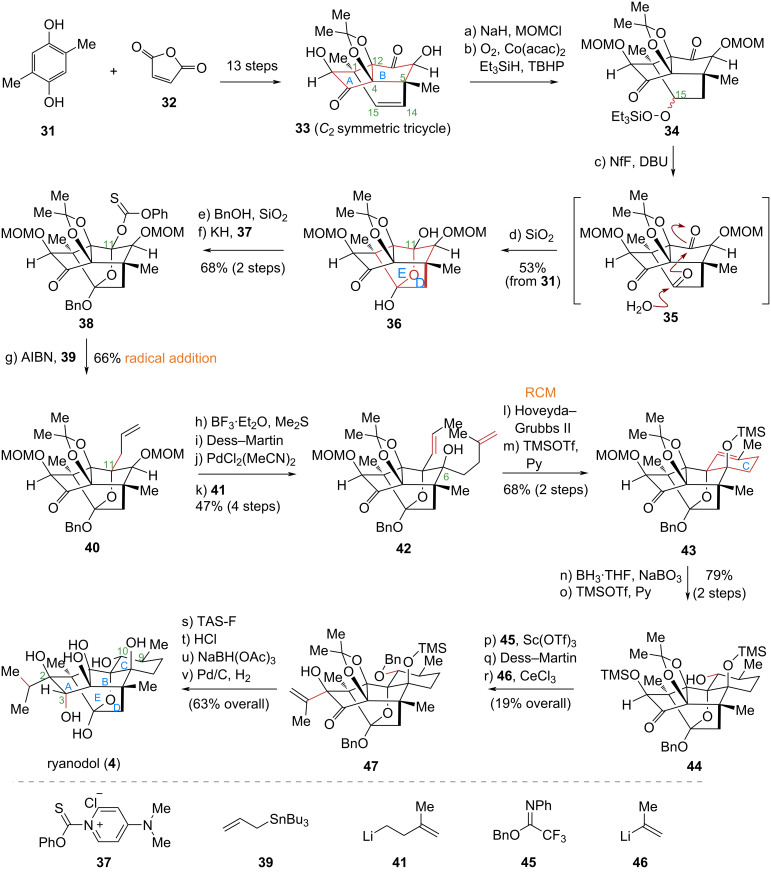
Inoue’s total synthesis of ryanodol (**4**).

In 2014, the Inoue group at the University of Tokyo reported a synthetic strategy for ryanodol (**4**) that leveraged substrate symmetry design, employing intramolecular radical coupling and olefin metathesis as key steps [46] (Scheme 4). Recognizing an embedded symmetric motif within the complex pentacyclic target, the authors designed a simplified C_2_-symmetric tricyclic intermediate, (±)-**33**, which was efficiently synthesized in 13 steps from commercial starting materials **31** and **32** by capitalizing on its molecular symmetry. A notable feature of this sequence was the simultaneous construction of four quaternary carbon centers (C1, C4, C5, and C12) and the core AB bicyclic skeleton, markedly improving synthetic efficiency. Subsequent oxidative desymmetrization of the C14–C15 olefin in (±)-**33** established the sterically hindered C11 quaternary carbon center. An α-alkoxy bridgehead radical addition then installed an allyl fragment, and ring-closing metathesis (RCM) smoothly formed the C ring to complete the core skeleton. The total synthesis was finalized by installing the four remaining stereocenters (C2, C3, C9, and C10).

The specific synthetic route is as follows: Commercially available compounds **31** and **32** were converted to the *C*_2_-symmetric **33** over 13 steps, enabling construction of the AB bicyclic skeleton in the target molecules. Compound **33** then underwent Mukaiyama hydration to adjust the C15 oxidation state, followed by water-promoted consecutive hemiacetalization to construct the oxa[3.2.1]-bridged ring system, thereby forming the D and E rings. Subsequently, the introduction of a tertiary hydroxy thiocarbonate at C11 afforded compound **38**. Under thermal conditions, **38** underwent smooth introduction of an allyl fragment via intermolecular radical addition reaction with allyltributylstannane, yielding compound **40**. After isomerization of the C11 allyl double bond and introduction of a C6 isobutenyl group, the resulting diene **42** underwent RCM catalyzed by the Hoveyda–Grubbs catalyst to form the pentacyclic skeleton **43**, thus completing the C ring of the natural product’s core structure. Finally, multisite functional group modifications and oxidation state adjustments enabled the total synthesis of ryanodol (**4**) in 35 steps.

**Scheme 5:**
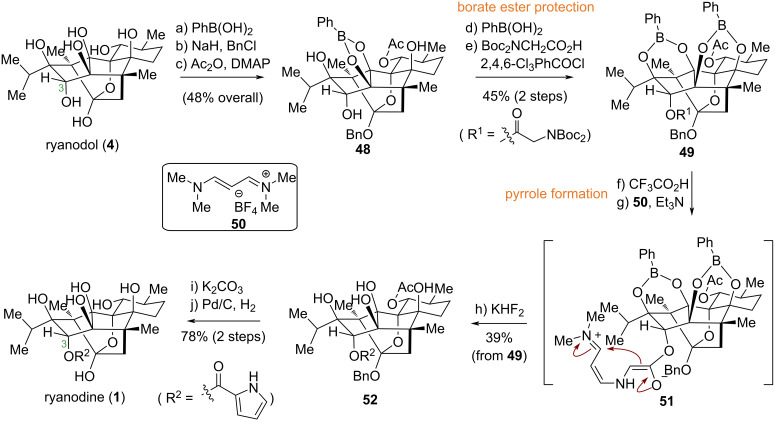
Inoue’s total synthesis of ryanodine (**1**) from ryanodol (**4**).

Ryanodine (**1**), a prominent member of the *Ryanoid* diterpene natural product family, exhibits remarkable insecticidal and pharmacological activities and serves as a potent modulator of intracellular calcium release channels. In contrast to ryanodol (**4**), compound **1** possesses a pyrrole-2-carboxylate ester moiety at the C3 position. This ester group can be cleaved via hydrolysis to yield **4**. However, the reverse transformation – the synthesis of ryanodine (**1**) from ryanodol (**4**) – had long eluded chemists. The primary challenge involved the selective installation of the bulky pyrrole unit onto the sterically congested C3 secondary hydroxy group within a polyfunctionalized, polyhydroxylated framework. In 2016, building upon prior work, the Inoue group reported the first total synthesis of ryanodine from ryanodol [47] (Scheme 5). Their strategy utilized a novel boronate protecting group to mask the four *syn*-oriented hydroxy groups. A critical step was the in-situ generation of the pyrrole-2-carboxylate unit from a glycine ester and 1,3-bis(dimethylamino)allylium tetrafluoroborate, which was then coupled to the C3 hydroxy group via Yamaguchi esterification. Global deprotection subsequently afforded ryanodine (**1**) in 10 steps, thus achieving this critical synthetic transformation.

**Scheme 6:**
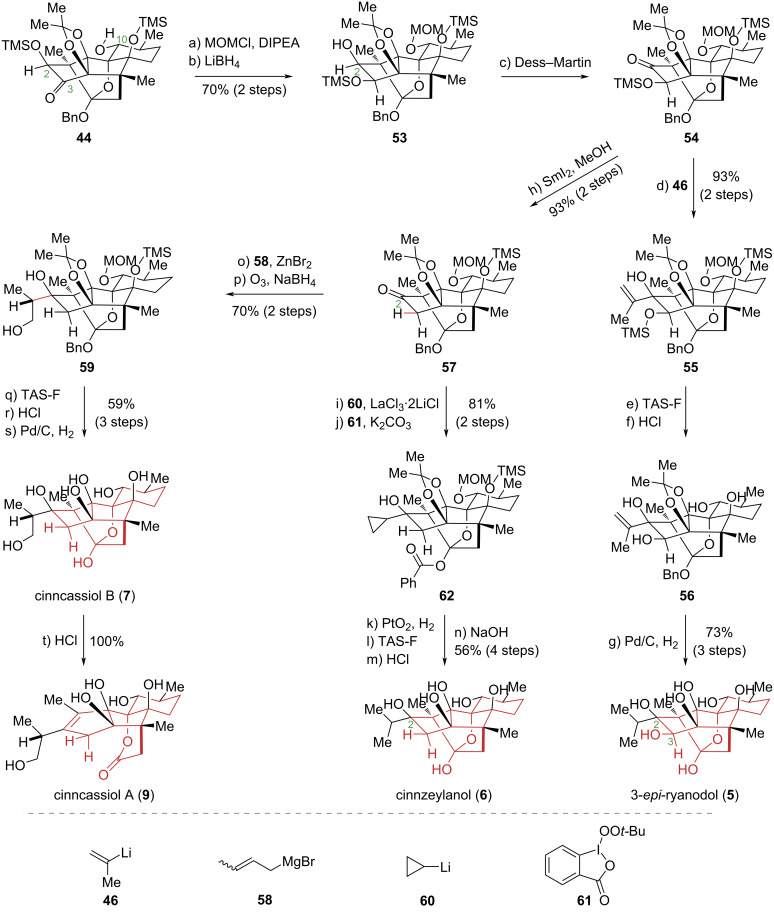
Inoue’s total synthesis of cinncassiol A (**9**), cinncassiol B (**7**), cinnzeylanol (**6**), and 3-*epi*-ryanodol (**5**).

Although the interaction of ryanodine with intracellular calcium release channels has been extensively studied, the mechanisms of action of other ryanoid diterpenoid natural products remain poorly understood. Elucidating the structure–activity relationships (SAR) of these compounds is essential for identifying the functional groups critical for their biological activity, thereby facilitating targeted molecular optimization. In 2016, the Inoue group accomplished the total synthesis of cinncassiol A (**9**) and B (**7**), cinnzeylanol (**6**), and 3-*epi*-ryanodol (**5**) through precisely controlled reactions with high stereoselectivity [15] (Scheme 6). Their approach allowed for the introduction of diverse substituents at the C2 position and precise modulation of oxidation states at other sites, including C3. The synthesis of 3-*epi*-ryanodol (**5**) commenced with compound **44**. After the protection of the C10 secondary hydroxy group, a sterically controlled, face-selective reduction of the C3 carbonyl, a silyl transform, and oxidation of the C2 secondary hydroxy group afforded intermediate **54**. This sequence successfully installed the C3 hydroxy group with the requisite stereochemistry for 3-*epi*-ryanodol (**5**). Subsequent introduction of an isopropyl group at C2 and global deprotection yielded the natural product. Similarly, starting from **57**, installation of an allyl group at C2, followed by oxidative cleavage, reduction, and deprotection, provided cinncassiol B (**7**). Subjecting this compound to an acid-promoted fragmentation reaction then completed the total synthesis of cinncassiol A (**9**). Furthermore, from intermediate **57**, the introduction of an isopropyl group at C2 and subsequent deprotection furnished cinnzeylanol (**6**).

#### Reisman’s total synthesis of (+)-ryanodine (**1**), (+)-20-deoxyspiganthine (**2**), and (+)-ryanodol (**4**)

**Scheme 7:**
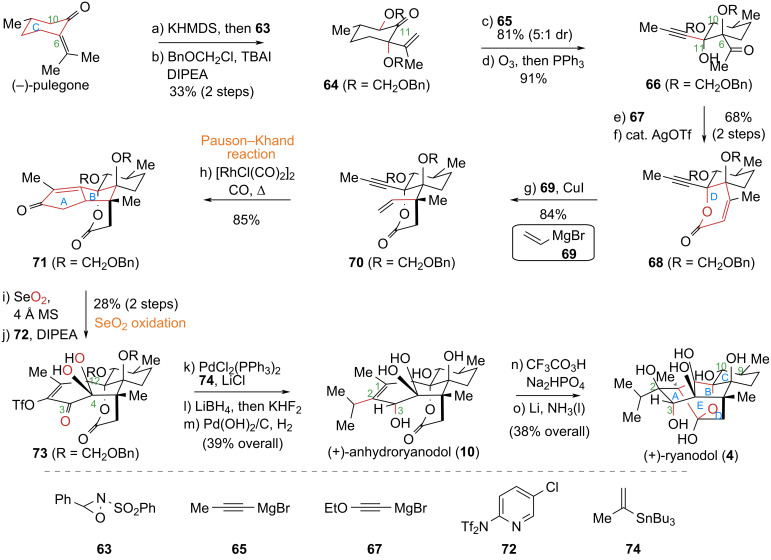
Reisman’s total synthesis of (+)-ryanodol (**4**).

In 2016, the Reisman group at Caltech reported an asymmetric total synthesis of (+)-ryanodol (**4**) in just 15 steps, highlighting the Pauson–Khand cyclization and a selenium dioxide-mediated selective oxidation as key transformations [48] (Scheme 7). To construct the multi-substituted five-membered ring in the target molecule, the authors strategically employed the Pauson–Khand reaction – a powerful method for building five-membered rings. This single [2 + 2 + 1] cycloaddition step efficiently converted a simple linear precursor into a complex bicyclic system. Subsequent late-stage modifications of the enone skeleton introduced multiple chiral centers, significantly enhancing overall synthetic efficiency. A further highlight of this work was the development of a selenium dioxide-mediated regioselective oxidation. Leveraging the existing chiral centers in the molecular framework, this strategy allowed for the simultaneous installation of the desired oxidation states at three distinct positions (C3, C4, C12) in a single step. This obviated the need for protecting groups and individual oxidation state adjustments, greatly streamlining the synthesis of this polyhydroxylated diterpene and underscoring the reaction’s utility in improving synthetic efficiency.

The specific synthetic route commenced from (−)-pulegone. After introducing oxidation states at C6 and C10 and installing an alkynyl group at C11, oxidative cleavage of a double bond yielded the key propargylic alcohol intermediate **66**. This compound underwent a 1,2-addition with alkynyl Grignard reagent **67**, and the resulting adduct was subjected to AgOTf-catalyzed lactonization to successfully construct the D ring of target molecular framework. Next, a 1,4-addition reaction introduced a vinyl group to compound **68**, affording compound **70**. A Pauson–Khand cyclization of **70** under [RhCl(CO)_2_]_2_/CO conditions smoothly furnished the ABCD tetracyclic core skeleton **71**. Treatment of **71** with SeO_2_ effected a multi-site sequential oxidation, and subsequent triflation yielded triflate **73**. Finally, compound **73** underwent a sequence of transformations: introduction of an isopropyl group at C2, directed reduction of the C3 carbonyl, epoxidation of the C1–C2 double bond, and Li/NH_3_-promoted reductive cyclization to construct the core E ring, completing the asymmetric total synthesis of (+)-ryanodol (**4**).

**Scheme 8:**
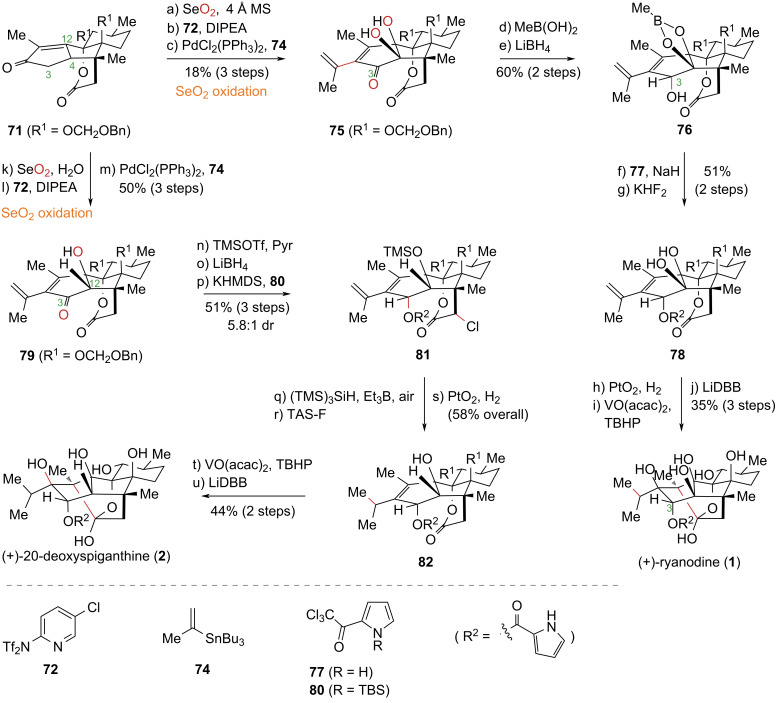
Reisman’s total synthesis of (+)-ryanodine (**1**) and (+)-20-deoxyspiganthine (**2**).

The 800-fold greater binding affinity of (+)-ryanodine (**1**) for cardiac ryanodine receptors (RyRs) compared to its hydrolysis product, (+)-ryanodol (**4**), indicates that the pyrrole-2-carboxylate unit at the C3 position is critical for receptor binding, as established by structure–activity relationship (SAR) studies [28]. However, the direct and selective modification of the highly sterically hindered C3 hydroxy group within this polyhydroxylated molecular framework has posed a significant synthetic challenge, impeding the preparation of derivatives for SAR exploration. In 2017, the Reisman group addressed this issue by drawing upon an analysis of Deslongchamps’s prior synthetic work [45] (Scheme 8). They hypothesized that utilizing an intermediate from the synthesis of anhydroryanodol (**10**), which features a less hindered C3 hydroxy group, would circumvent the chemoselectivity problems. Their strategy involved first installing the pyrrole carboxylate unit on this more accessible position, followed by constructing the ketal moiety of the ryanodine skeleton via an established single-electron reductive cyclization to complete the total synthesis. This strategic inversion of the synthetic sequence enabled direct acylation of anhydroryanodol derivatives, facilitating the introduction of the key pyrrole-2-carboxylate unit. This method effectively resolved a major obstacle in the synthesis of ryanodine (**1**) and established a versatile approach for introducing diverse C3 ester substituents for future SAR studies [49].

The synthesis commenced from advanced intermediate **71** (from their prior work). Sequential SeO_2_-mediated oxidation, triflation, and introduction of an isopropenyl group afforded compound **75**. Subsequent protection of the vicinal diol as a boronic ester and diastereoselective reduction of the C3 carbonyl group yielded compound **76**. Esterification with acylating reagent **77** under basic conditions, followed by boronic ester removal, provided compound **78**. Finally, a sequence comprising terminal alkene reduction, epoxidation of the tetrasubstituted alkene, and LiDBB-promoted intramolecular reductive cyclization and deprotection completed the asymmetric total synthesis of (+)-ryanodine (**1**) in 17 steps. Notably, the additive used in the SeO_2_ oxidation critically influenced the reaction outcome [50–51]. Employing 4 Å molecular sieves afforded product **75** with oxidation states installed at both the C4 and C12 positions. In contrast, using H_2_O as an additive yielded product **79**, bearing a single oxidation state at the C12 position. Leveraging this regioselective oxidation, the authors achieved the total synthesis of (+)-20-deoxyspiganthine (**2**) from compound **71**. Thus, **71** was converted to **79** via selective SeO_2_ oxidation (with H_2_O), triflation, and isopropenyl installation. After protecting the C12 tertiary alcohol and performing a diastereoselective reduction of the C3 ketone, the acyl pyrrole group was introduced to yield **81**. During acyl pyrrole installation, excess KHMDS enolized the lactone to suppress lactone-C3 hydroxy transesterification. However, α-chlorination of the ester carbonyl was unavoidable. Finally, reductive dechlorination, terminal alkene reduction, and intramolecular reductive cyclization culminated in the completion of the first asymmetric total synthesis of (+)-20-deoxyspiganthine (**2**) in 19 steps.

#### Micalizio’s formal total synthesis of ryanodol (**4**)

**Scheme 9:**
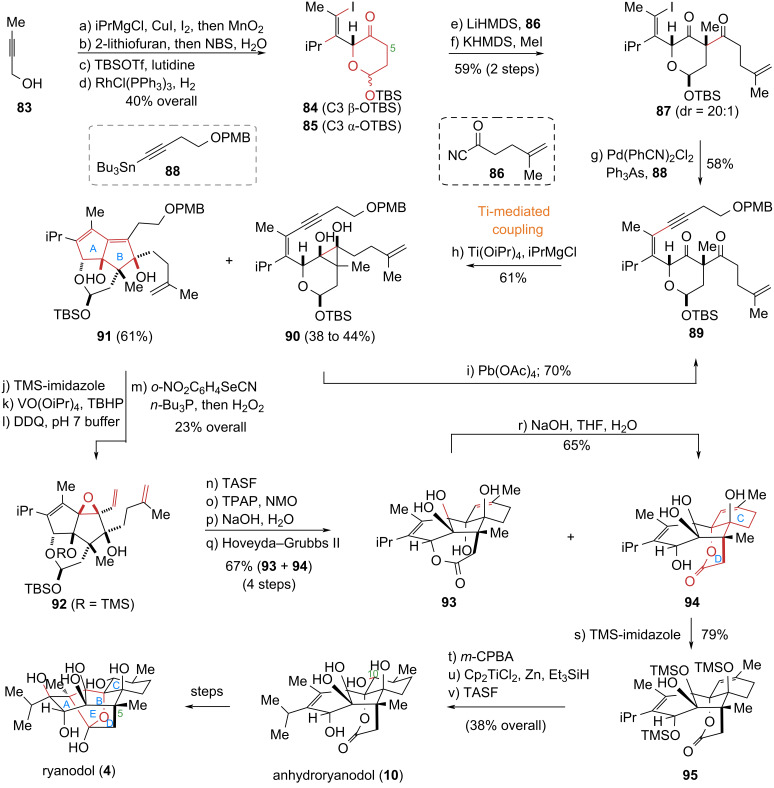
Micalizio’s formal total synthesis of ryanodol (**4**).

In 2020, the Micalizio group at the University of California, San Diego, achieved the total synthesis of anhydroryanodol (**10**) and a formal synthesis of ryanodol (**4**) through a key low-valent titanium-mediated intramolecular stereoselective coupling of alkynes with 1,3-dicarbonyl compounds [52] (Scheme 9). To construct the oxygenated fused-ring system with contiguous stereocenters characteristic of the target molecule, the authors strategically implemented this methodology. This approach efficiently established two carbon–carbon and four carbon–oxygen bonds while introducing four contiguous stereocenters, successfully assembling the highly functionalized AB ring system. This work not only demonstrates the efficacy of the titanium-mediated intramolecular alkyne-1,3-diketone coupling but also provides a novel strategic approach for synthesizing natural products within this structural class.

The synthesis commenced from commercially available compound **83**. Sequential alkyne difunctionalization, furyl group installation, Achmatowicz rearrangement, and subsequent functional group manipulations provided intermediates **84** and **85**. C5-acylation and methylation under kinetically controlled conditions followed by Sonogashira coupling yielded cyclization precursor **89**. Treatment of **89** with Ti(OiPr)_4_/iPrMgCl promoted the intramolecular stereoselective alkyne–1,3-dicarbonyl coupling, resulting in the construction of the AB ring system. This transformation afforded tricyclic compound **91** as the major product, accompanied by minor amounts of by-product **90**. Subjecting **91** to epoxidation of the tetrasubstituted alkene followed by Grieco elimination yielded diene **92**. Subsequent oxidation of the hemiacetal, saponification of the lactone, intramolecular epoxide opening, and Hoveyda–Grubbs (II)-catalyzed RCM afforded tetracyclic compounds **94** and its transesterification product **93**, thus establishing the core C and D rings. Base-mediated equilibration fully converted **93** into lactone **94**. Finally, selective hydroxy protection in **94**, diastereoselective introduction of the C10 secondary alcohol, and global deprotection completed the total synthesis of anhydroryanodol (**10**). Application of established Deslongchamps and Reisman protocols then enabled the formal synthesis of ryanodol (**4**) in 22 steps.

#### Zhao’s total synthesis of garajonone (**8**) and formal syntheses of ryanodol (**4**) and ryanodine (**1**)

**Scheme 10:**
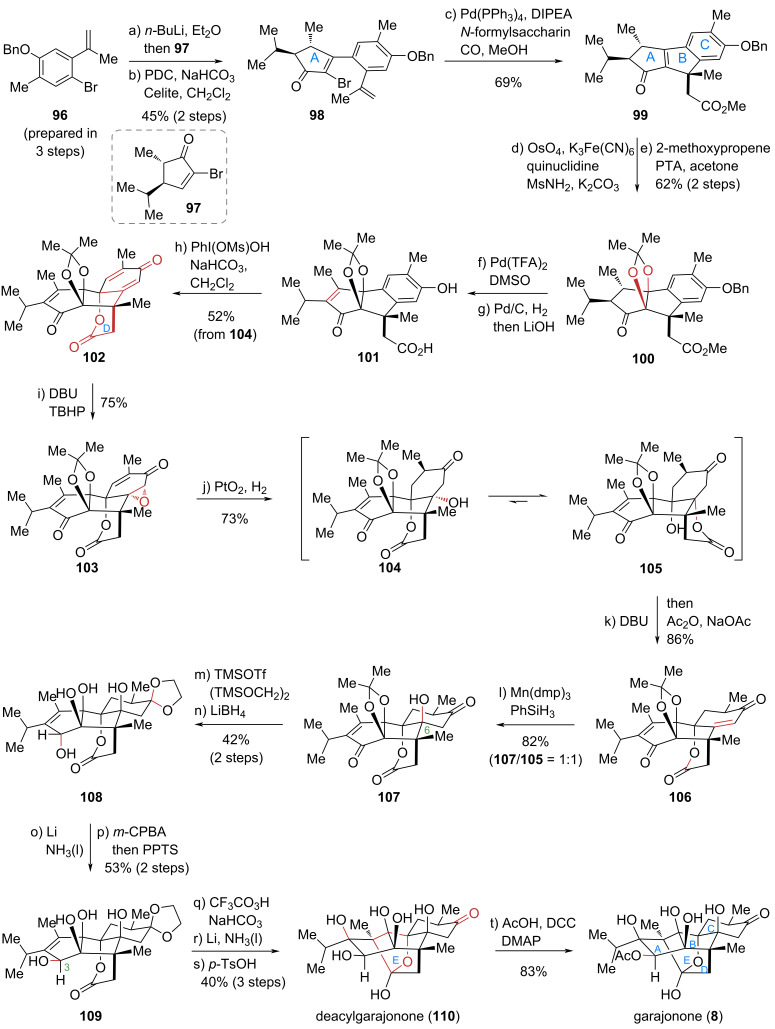
Zhao’s total synthesis of garajonone (**8**).

Traditional total synthesis strategies often follow a linear, stepwise approach – analogous to “climbing a staircase” – where each successive step involves functional group interconversions and protecting group manipulations, culminating in low overall efficiency. In contrast, Baran’s “two-phase” synthesis strategy emulates nature’s “cyclization–functionalization” logic [53–54]. This approach is akin to “taking an elevator”, prioritizing the rapid assembly of the molecular core skeleton before undertaking precise late-stage functionalization. This strategy has proven highly successful for synthesizing complex terpenoids, as exemplified by the Baran group’s 2013 total synthesis of ingenol [55]. Typically, the biosynthesis of polycyclic diterpenes occurs in two distinct phases: an initial cyclase-mediated cyclization phase to form the carbon framework, followed by an oxidase-catalyzed phase to install the requisite oxidation states. Inspired by this general biosynthetic pathway, the Zhao group employed a similar two-phase strategy to achieve the first total synthesis of the *Ryania* diterpenoid garajonone (**8**) in 2025 [56] (Scheme 10).

Key achievements of this synthesis include: (1) application of palladium-catalyzed Heck/carbonylative cascade cyclization to efficiently construct the core tricyclic carbon skeleton, and (2) systematic oxidation state manipulation of this scaffold to precisely introduce its dense array of oxygenated stereocenters. The construction of the C6 quaternary stereocenter represented a particularly formidable challenge in the late stage. This was successfully accomplished via an epoxide ring-opening/tandem lactonization/olefin hydration sequence. In total, 12 consecutive redox manipulations (7 oxidations and 5 reductions) established all stereocenters, with subsequent functional group transformations completing the total synthesis of garajonone (**8**).

The synthesis commenced with the preparation of key cyclization precursor **98** via Barbier coupling and Babler–Dauben oxidative rearrangement. A pivotal palladium-catalyzed Heck/carbonylative cyclization then efficiently furnished the ABC tricyclic core **99**. Notably, adding *N*-formylsaccharin under a CO atmosphere significantly suppressed side reactions, yielding the cyclized product in excellent yield and selectivity. This indicates that *N*-formylsaccharin, beyond acting as a CO-releasing agent, may function as a ligand in the catalytic cycle to regulate the palladium catalyst’s activity and stability. While its mechanism remains incompletely understood, this additive’s unique efficacy in such transformations is unprecedented. Subsequent steps involved hydroxylation of the double bond and the protection of the vicinal diol as a dimethyl ketal giving ester **100**. Oxidative dehydrogenation, benzyl deprotection and ester hydrolysis produced carboxylic acid **101**, which upon oxidative dearomatization yielded dienone **102**, thus completing the D-ring. Regioselective epoxidation to **103** and reduction (using Adams' catalyst) through intermediate **104** gave lactone **105**. A retro-*oxa*-Michael/intramolecular transesterification sequence produced mono-enone **106**, whose hydration installed the C6 stereocenter to yield **107**. Further protecting group and oxidation state adjustments afforded lactone **108**, which was transformed via an intramolecular S_N_2′ reaction (single-electron reduction), *m*-CPBA epoxidation, acid-promoted fragmentation, and face-selective hydroxylation at C3 to yield **109**. A final sequence of epoxidation, single-electron reductive cyclization, and ethylene glycol deprotection delivered hemiketal **110**, completing the E-ring formation. Finally, selective acetylation of the secondary hydroxy group culminated in the first total synthesis of garajonone (**8**) in 20 steps.

**Scheme 11:**
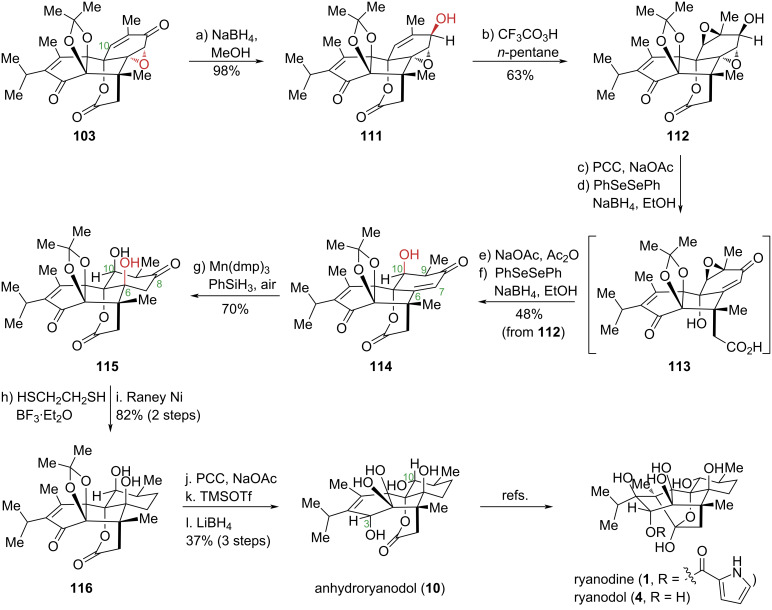
Zhao’s formal total synthesis of ryanodol (**4**) and ryanodine (**1**).

The structural diversity of ryanodine-type diterpenoid natural products arises primarily from variations in oxidation patterns and stereochemical configurations, particularly at the C3, C8, and C10 positions. These subtle structural differences present substantial challenges in developing a unified synthetic strategy capable of accessing diverse members of this family. The Zhao group recently achieved the first total synthesis of the *Ryania* diterpenoid garajonone (**8**) and its epimer 3-*epi*-garajonone. Unlike the representative ryanodine diterpenoids and analogs prepared by Inoue, Reisman, and Micalizio, these compounds feature oxidation at the C8 position instead of the more conventional C10 site. Capitalizing on this oxidative divergence, they investigated whether a common advanced intermediate could be selectively functionalized to install either C8 or C10 oxidation, followed by subsequent oxidation state adjustments and functional group manipulations to accomplish the total synthesis of the target natural products [57] (Scheme 11). Based on retrosynthetic analysis of previous routes, they employed common intermediate **103** as the starting material. Key transformations included regioselective and stereoselective alkene epoxidation, organoselenium-mediated reductive cleavage of the α,β-epoxy ketone, and a hydroxy-directed stereospecific Mukaiyama hydration. These operations successfully introduced the C6 and C10 oxidation states, enabling the synthesis of the representative *Ryania* diterpenoid degradation product anhydroryanodol (**10**) and the formal total syntheses of ryanodol (**4**) and ryanodine (**1**). This work establishes the first unified synthetic approach for ryanodine-type diterpenoids with varying oxidation patterns and provides a robust platform for synthesizing other family members and their structural analogues.

The synthetic sequence commenced with common intermediate **103** as the starting material. Stereoselective reduction of **103** yielded compound **111** as a single diastereomer. Systematic optimization of reaction conditions revealed that oxidation with freshly prepared trifluoroperacetic acid in *n*-pentane converted **111** to bis-epoxide **112** with excellent stereoselectivity and yield. Subjecting **112** to oxidation and organoselenium-mediated regioselective α,β-epoxy ketone opening, followed by intramolecular transesterification and elimination, provided carboxylic acid **113** in a single operation. Subsequent activation of **113** with acetic anhydride, relactonization, and organoselenium-mediated regioselective epoxide opening yielded mono-enone **114**, successfully installing the key C9 and C10 chiral centers with the required oxidation states. Notably, the bis-epoxy ketone exhibited distinct reactivity under ring-opening conditions compared to mono-epoxy substrates, presumably due to steric constraints. Leveraging the directing ability of the C10 hydroxy group, stereospecific Mukaiyama hydration of the C6–C7 double bond was achieved, furnishing compound **115** and establishing the correct C6 configuration. This transformation represents the first reported example of a hydroxy-directed Mukaiyama hydration reaction. The C8 carbonyl group was then protected as its 1,3-dithiolane derivative by treatment with 1,2-ethanedithiol. Without purification, the resulting intermediate was directly subjected to Raney nickel desulfurization, reducing the C8 carbonyl to a methylene group and delivering compound **116**. Finally, oxidation of the secondary alcohol, dimethyl ketal deprotection, and hydroxy-directed reduction installed the C3 hydroxy group and inverted the C10 stereochemistry, thereby completing the total synthesis of anhydroryanodol (**10**). By applying established strategies developed by Deslongchamps and Reisman to this intermediate, they enabled the formal total syntheses of ryanodol (**4**) and ryanodine (**1**).

## Summary and Outlook

*Ryania* diterpenoid natural products continue to attract considerable research interest due to their intricate chemical architectures and distinctive biological properties. Through decades of dedicated effort, synthetic chemists have accomplished the total synthesis of several members within this family that share a common core scaffold yet exhibit diverse oxidation patterns and stereochemical configurations, with ryanodine (**1**) representing a landmark example (Table 1).

**Table 1:** The total synthesis of *Ryania* diterpenoids (1979–2025).

NPs^a^	Research group	Year	Key strategy/steps

ryanodol	Deslongchamps	1979	• Diels–Alder reaction • intramolecular aldol • transannular aldol • reductive cyclization
	Inoue	2014	• desymmetric strategy • Mukaiyama hydration • bridgehead radical addition • ring-closing metathesis
	Reisman	2016	• chiral pool strategy • intramolecular Pauson–Khand • SeO_2_-mediated regioselective oxidation • reductive cyclization
	Micalizio	2020	• formal synthesis • Ti-mediated coupling • selective epoxy opening • ring-closing methathesis
	Zhao	2025	• formal synthesis • Pd-catalyzed Heck/carbonylative cascade • oxidative dearomatization • directed Mukaiyama hydration
3-*epi*-ryanodol	Deslongchamps	1993	• Diels–Alder reaction • intramolecular aldol • transannular aldol • reductive cyclization • epoxidation/fragmentation cascade
	Inoue	2016	• desymmetric strategy • Mukaiyama hydration • bridgehead radical addition • ring-closing metathesis
3-*epi*-ryanodine	Deslongchamps	1993	• Diels–Alder reaction • intramolecular aldol • transannular aldol • reductive cyclization • epoxidation/fragmentation cascade • late-stage pyrrole-2-carboxylate formation
ryanodine	Inoue	2016	• desymmetric strategy • Mukaiyama hydration • bridgehead radical addition • ring-closing metathesis • borate ester protection • in-situ pyrrole formation
	Reisman	2017	• chiral pool strategy • intramolecular Pauson–Khand • SeO_2_-mediated regioselective oxidation • early-stage pyrrole-2-carboxylate formation • reductive cyclization
cinnzeylanol	Inoue	2016	• desymmetric strategy • Mukaiyama hydration • radical addition • ring-closing metathesis • introduction of isopropyl
cinncassiol A	Inoue	2016	• desymmetric strategy • Mukaiyama hydration • radical addition • ring-closing metathesis • selective 1,2-addition
cinncassiol B	Inoue	2016	• desymmetric strategy • Mukaiyama hydration • radical addition • ring-closing metathesis • selective 1,2-addition
20-deoxyspiganthine	Reisman	2017	• chiral pool strategy • intramolecular Pauson–Khand • SeO_2_-mediated regioselective oxidation • early-stage pyrrole-2-carboxylate formation • reductive cyclization
garajonone	Zhao	2025	• two-phase strategy • Pd-catalyzed Heck/carbonylative cascade • oxidative dearomatization • selective redox • reductive cyclization

^a^NPs = natural product or its epimer.

This review has highlighted synthetic investigations of *Ryania* diterpenoids by various research groups, encompassing brief discussions of their isolation, structural elucidation, and biological activities. Particular emphasis has been placed on analyzing the strategic designs and key synthetic transformations employed in existing total syntheses, aiming to provide readers with a comprehensive overview of the current state of total synthesis achievements for this natural product family while stimulating further innovation in synthetic methodology. Evidently, the synthesis of *Ryania* diterpenoids remains one of the most formidable challenges in contemporary synthetic chemistry. The development of more efficient and broadly applicable synthetic strategies leveraging these complex molecular architectures continues to be a primary objective for synthetic chemists. Achieving this goal will require persistent innovation to transcend conventional synthetic paradigms and advance synthetic methods toward enhanced efficiency, precision, and sustainability. Concurrently, biological investigations of this natural product family remain relatively underdeveloped. Systematic evaluation of biological activities and structure–activity relationships, along with deeper exploration of the potential biological functions and practical applications of highly oxidized *Ryania* diterpenoids, will constitute crucial directions for future research.

## Data Availability

Data sharing is not applicable as no new data was generated or analyzed in this study.
